# MicroRNAs as Potential Biomarkers in the Differential Diagnosis of Lipomatous Tumors and Their Mimics

**DOI:** 10.3390/ijms23147804

**Published:** 2022-07-15

**Authors:** Hui Min Tan, He Cheng, Yew Chung Tang, Sai Mun Leong, Poh Yin Teo, Chi Kuen Lee, Victor Kwan Min Lee, Susan Swee-Shan Hue

**Affiliations:** 1 Department of Pathology, National University Hospital, National University Health System, Singapore 119074, Singapore; patthm@nus.edu.sg (H.M.T.); patvlkm@nus.edu.sg (V.K.M.L.); 2 MiRXES Lab, Singapore 138623, Singapore; hecheng@mirxes.com (H.C.); yewchungtang@mirxes.com (Y.C.T.); 3 Department of Pathology, Yong Loo Lin School of Medicine, National University of Singapore, Singapore 117597, Singapore; patlsm@nus.edu.sg (S.M.L.); patleeck@nus.edu.sg (C.K.L.); 4 Division of Oncology Pharmacy, National University Cancer Institute Singapore, National University Health System, Singapore 119074, Singapore; poh_yin_teo@nuhs.edu.sg; 5 Advanced Molecular Pathology Laboratory, Institute of Molecular and Cell Biology, Agency for Science, Technology and Research (A*STAR), Singapore 138673, Singapore

**Keywords:** microRNA, lipoma, well-differentiated liposarcoma, dedifferentiated liposarcoma, undifferentiated pleomorphic sarcoma

## Abstract

Adipocytic tumors are the most common subtype of soft tissue tumors. In current clinical practice, distinguishing benign lipomas from well-differentiated liposarcomas (WDLPS), as well as dedifferentiated liposarcomas (DDLPS) from their morphologic mimics, remains a significant diagnostic challenge. This is especially so when examining small biopsy samples and without the aid of additional ancillary tests. Recognizing the important role that microRNAs (miRNAs) play in tumorigenesis and their potential utility in tumor classification, we analyzed routine clinical tissue samples of benign and malignant lipomatous tumors, as well as other sarcoma mimics, to identify distinguishing miRNA-based signatures that can aid in the differential diagnosis of these entities. We discovered a 6-miRNA signature that separated lipomas from WDLPS with high confidence (AUC of 0.963), as well as a separate 6-miRNA signature that distinguished DDLPS from their more aggressive histologic mimics (AUC of 0.740). Functional enrichment analysis unveiled possible mechanistic involvement of these predictive miRNAs in adipocytic cancer-related biological processes and pathways such as PI3K/AKT/mTOR and MAPK signaling, further supporting the relevance of these miRNAs as biomarkers for adipocytic tumors. Our results demonstrate that miRNA expression profiling may potentially be used as an adjunctive tool for the diagnosis of benign and malignant adipocytic tumors. Further validation studies are warranted.

## 1. Introduction

Soft tissue tumors (STT) represent a heterogeneous and biologically diverse group of mesenchymal tumors with highly varied clinical behavior and treatment approaches. The current standard for diagnosis is based primarily on histopathologic evaluation. However, due to their relative rarity and overlapping morpho-phenotypic features, making an accurate diagnosis of STT is challenging, even for experienced surgical pathologists. With the recent discovery of tumor-specific genetic alterations in a growing number of STTs, molecular testing has become increasingly indispensable as an adjunct to the diagnosis of these neoplasms.

Adipocytic tumors are the most common type of STT [1], with lipomas and liposarcomas representing the most common benign and malignant STTs, respectively. Benign lipomas and their variants can be cured by simple surgical excision, and recurrences are rare. Of the liposarcoma subtypes, atypical lipomatous tumors/well-differentiated liposarcomas (collectively termed WDLPS) constitute the most common subtype, comprising 40% of all liposarcomas [2]. WDLPS is locally aggressive and can undergo dedifferentiation to a higher-grade sarcoma (i.e., dedifferentiated liposarcoma (DDLPS)) that has significant metastatic potential. Given the distinct biological nature of these lipomatous neoplasms, accurate diagnosis is hence necessary for optimal management. 

In current clinical practice, the diagnosis of lipomatous tumors is based primarily on histomorphologic features which may not always be clear cut. For example, lipomas with degenerative changes can show considerable variation in adipocytic cell size, nuclear atypia and fat necrosis and similar changes can also be seen in WDLPS. Conversely, the diagnostic features of WDLPS may be absent or under-represented in small tissue samples, which are becoming commoner as clinicians gravitate towards less invasive means to obtain diagnostic tissue. Another area of diagnostic challenge is in separating DDLPS from other high-grade sarcomas. DDLPS have a wide morphologic spectrum but most frequently manifest as pleomorphic or spindle cell sarcomas resembling myxofibrosarcoma (MFS) and/or undifferentiated pleomorphic sarcoma (UPS). Given that DDLPS has a much lower metastatic rate (15–20%) as compared to the other two sarcoma subtypes (35–50%) [2], their separation is necessary to facilitate appropriate treatment. Hence, in the above instances, adjunctive tools such as fluorescent in situ hybridization (FISH) and immunohistochemistry have to be employed to aid the pathologist in making the diagnosis, but they may not always be readily available or definitive in their interpretation. 

MicroRNAs (miRNAs) belong to a family of evolutionarily conserved, short non-coding RNAs of approximately 16–26 nucleotides in length involved in post-transcriptional modulation of gene expression [3,4]. They play vital regulatory roles in various cellular processes such as proliferation, differentiation, and apoptosis by degrading their target messenger RNAs (mRNAs) or repressing translation [5,6]. In the realm of cancer pathophysiology, miRNA dysregulation has been widely linked to the development and progression of numerous tumor types [7,8]. Given their roles in oncogenesis, distinct expression profiles, and high stability in routine clinical samples [9], miRNA expression profiling has immense potential to serve as a clinical diagnostic tool to improve tumor classification and subtyping. 

Hence, with the objective of exploring the potential diagnostic utility of miRNAs, we performed miRNA expression profiling on formalin-fixed paraffin-embedded tissue samples of lipomas, WDLPS, DDLPS, MFS, and UPS using a novel quantitative polymerase chain reaction (qPCR) miRNA assay, aiming to look for distinctive miRNA signatures that can be used as a diagnostic adjunct in clinical practice to facilitate the classification of these tumors, comparing their accuracy against the current diagnostic gold standard which is histomorphology. 

## 2. Results

### 2.1. Diagnostic Challenges of Lipomatous Tumors

#### 2.1.1. Well-Differentiated Lipomatous Tumor

**Figure 1:**
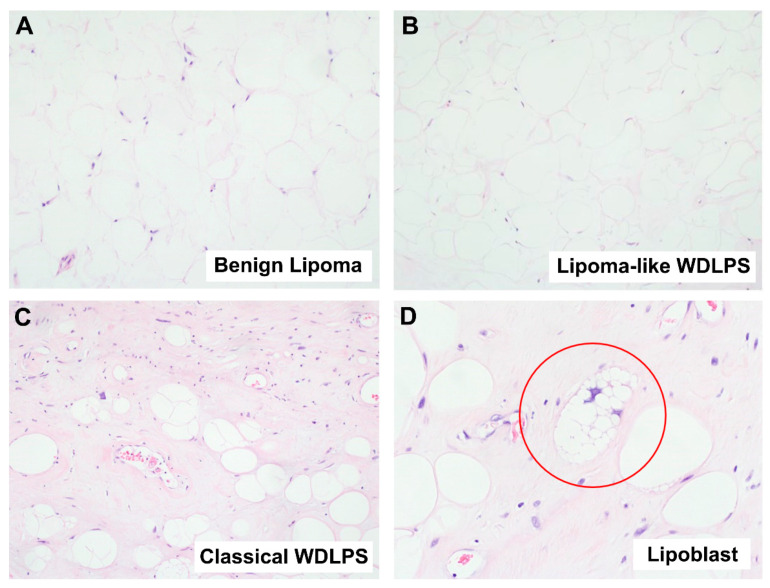
Histological comparison between benign lipoma and malignant well-differentiated liposarcoma (WDLPS). (**A**) Microscopic image of a benign lipoma, showing lobules of mature adipocytes with minimal variation in adipocytic size and no nuclear atypia (Hematoxylin and Eosin (H&E) stain, 100× magnification). (**B**) Microscopic image showing lipoma-like WDLPS (H&E stain, 100× magnification). A lipoma-like WDLPS can show marked morphologic similarity to lipoma (Figure 1A), making it hard to differentiate between the two based on morphology alone. (**C**) Microscopic image of a malignant WDLPS, showing adipocytes with significant variation in size and shape as well as enlarged, hyperchromatic nuclei (H&E stain, 100× magnification). (**D**) Microscopic image of a lipoblast (circled in red) from a case of malignant WDLPS (H&E stain, 200× magnification). A lipoblast is characterized by multiple clear cytoplasmic vacuoles that identify a hyperchromatic nucleus.

A typical case of benign lipoma is histologically characterized by lobules of mature adipocytes with minimal variation in adipocytic size and no nuclear atypia (Figure 1A). On the other hand, a classic case of a malignant WDLPS is histologically characterized by adipocytes with significant variation in size and shape, as well as enlarged hyperchromatic nuclei (Figure 1C). Stromal cells may also appear multi-nucleated. In some cases, lipoblasts can be seen (Figure 1D). However, the diagnostic challenge arises when an otherwise bland-appearing lipomatous neoplasm has clinical or radiological features that raise the suspicion of a malignant lipomatous neoplasm, for instance, large size or deep location. In these cases, the possibility of a lipoma-like WDLPS (Figure 1B) has to be considered and histomorphology alone is unable to differentiate this entity from a benign lipoma (Figure 1A). Hence, detection of *MDM2* amplification via FISH test is required, and doing so will incur a higher cost of diagnosis.

#### 2.1.2. Dedifferentiated Lipomatous Tumor

**Figure 2:**
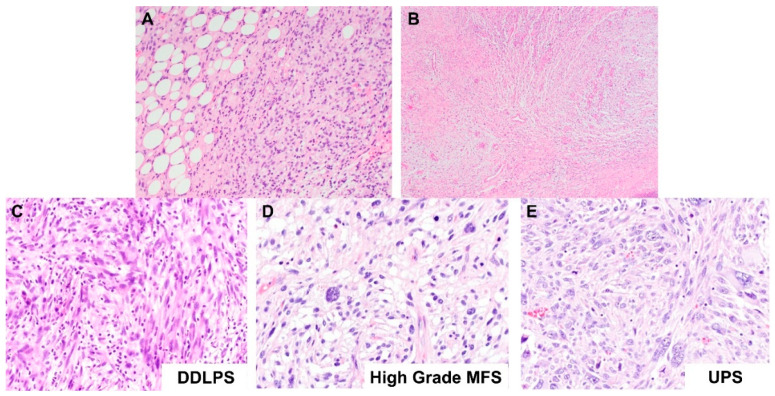
Histological comparison between dedifferentiated liposarcoma (DDLPS), myxofibrosarcoma (MFS) and undifferentiated pleomorphic sarcoma (UPS). (**A**) Microscopic image of a DDLPS (H&E stain, 100× magnification). The adjacent well-differentiated component is identified (left aspect), which serves as the diagnostic area for DDLPS. (**B**) Microscopic image of an MFS (H&E stain, 40× magnification). The image shows the myxoid low-grade area of MFS, characterized by curvilinear blood vessels, spindle cells, lobulated areas and myxoid stroma. This area serves as the diagnostic area for high-grade MFS. (**C**–**E**) Microscopic images showing side-by-side comparison of (**C**) DDLPS, (**D**) high-grade MFS and (**E**) UPS (H&E stain, 200× magnification). As illustrated here, the three pleomorphic sarcomas exhibit similar pleomorphic morphology, making the differential diagnosis of the three tumor subtypes difficult.

The histogenesis or lineage of a high-grade pleomorphic-appearing sarcoma can generally be determined if the lower grade component can be identified histologically. For example, DDLPS can be diagnosed histologically when an otherwise undifferentiated-appearing pleomorphic sarcoma shows areas of lipogenic differentiation typical of a WDLPS (Figure 2A). In these cases, if the clinical context is concordant, the diagnosis of DDLPS can be rendered without the need for further ancillary tests. Similarly, high-grade MFS can be diagnosed histologically when the myxoid low-grade area of MFS, characterized by curvilinear blood vessels, spindle cells, lobulated areas and myxoid stroma, is seen (Figure 2B). 

However, sometimes, the lipogenic component of a DDLPS can be very focally present or even absent, such that we do not see it in our histologic sections. In these instances, the dedifferentiated component of the DDLPS (Figure 2C) may show significant morphologic overlap with high-grade MFS (Figure 2D) and UPS (Figure 2E), exhibiting pleomorphic morphology characterized by significant variation in shapes and sizes of cells, hypercellularity, mitotic figures, hyperchromatic nuclei, atypical stromal cells, and in some cases, tumor necrosis. This overlapping pleomorphic morphology makes the differential diagnosis of the three tumor subtypes difficult, especially in the event of small biopsies, where the diagnostic areas of the tumor are not captured due to limited sampling of the tumor. This conundrum is also further complicated by the fact that these three tumors do not have specific or diagnostic immunohistochemical expression profiles as well.

### 2.2. miRNAs Expression Profiles Discriminate Benign Lipoma from Liposarcoma (WDLPS and DDLPS) and Other Sarcoma Subtypes (MFS and UPS)

**Figure 3:**
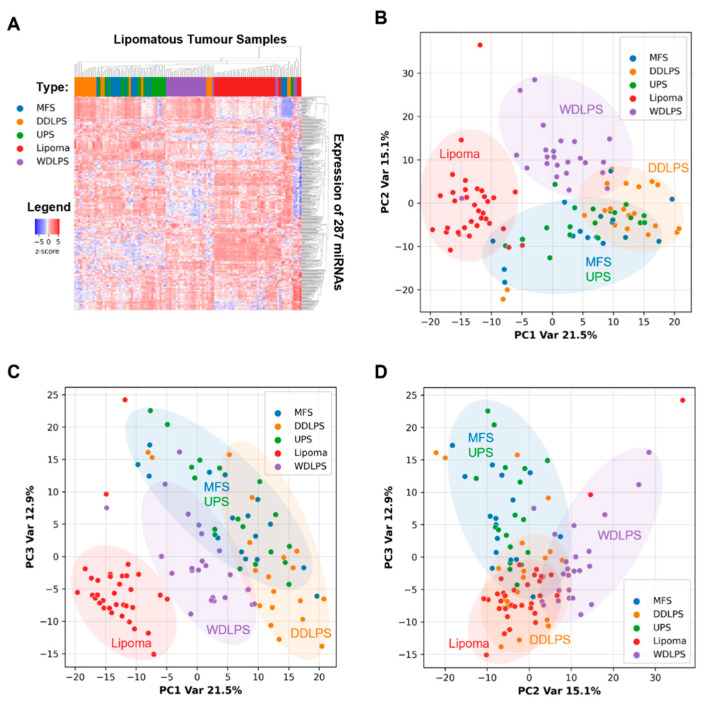
Biologically distinct soft tissue tumors can be differentiated by microRNA expression profiles. (**A**) Heatmap showing hierarchical clustering of benign (lipoma) and malignant (WDLPS, DDLPS, MFS, UPS) soft tissue tumor FFPE samples using their microRNA expression profiles measured by qPCR. (**B**–**D**) Results of principal component analysis (PCA) on miRNA expression profiles of soft tissue tumor FFPE samples shown in graphs plotting (**B**) principal component 2 (PC2) against principal component 1 (PC1), (**C**) principal component 3 (PC3) against PC1, and (**D**) PC3 against PC2.

To explore if miRNA biomarkers have diagnostic utility in discriminating benign lipoma from liposarcoma and other sarcoma subtypes, expression levels of 350 miRNAs were measured in a total of 112 FFPE tissue samples from lipoma, WDLPS, DDLPS, MFS, and UPS cases. Out of the 350 miRNAs measured, 287 miRNAs were detected in more than 90% of all tissue samples. Hierarchical clustering performed using the expression of these 287 miRNAs resulted in clusters representing the majority of lipoma, WDLPS, DDLPS, and a mix of MFS/UPS samples (Figure 3A). Principal component analysis showed that the first three principal components (PC1, PC2, and PC3) captured 21.5%, 15.1%, and 12.9% of the variation in miRNA expression profiles, respectively. Lipoma can be well discriminated from WDLPS, DDLPS, and MFS/UPS through variation in miRNA expression profiles captured in PC1 and PC2 (Figure 3B) or PC1 and PC3 (Figure 3C). Similarly, WDLPS, DDLPS, and MFS/UPS samples could be partially differentiated from each other through the variation in miRNA expression profiles captured in the first three principal components (Figure 3B–D). 

### 2.3. Expression Levels of Six miRNAs Distinguish Lipoma from WDLPS with High Accuracy

**Figure 4:**
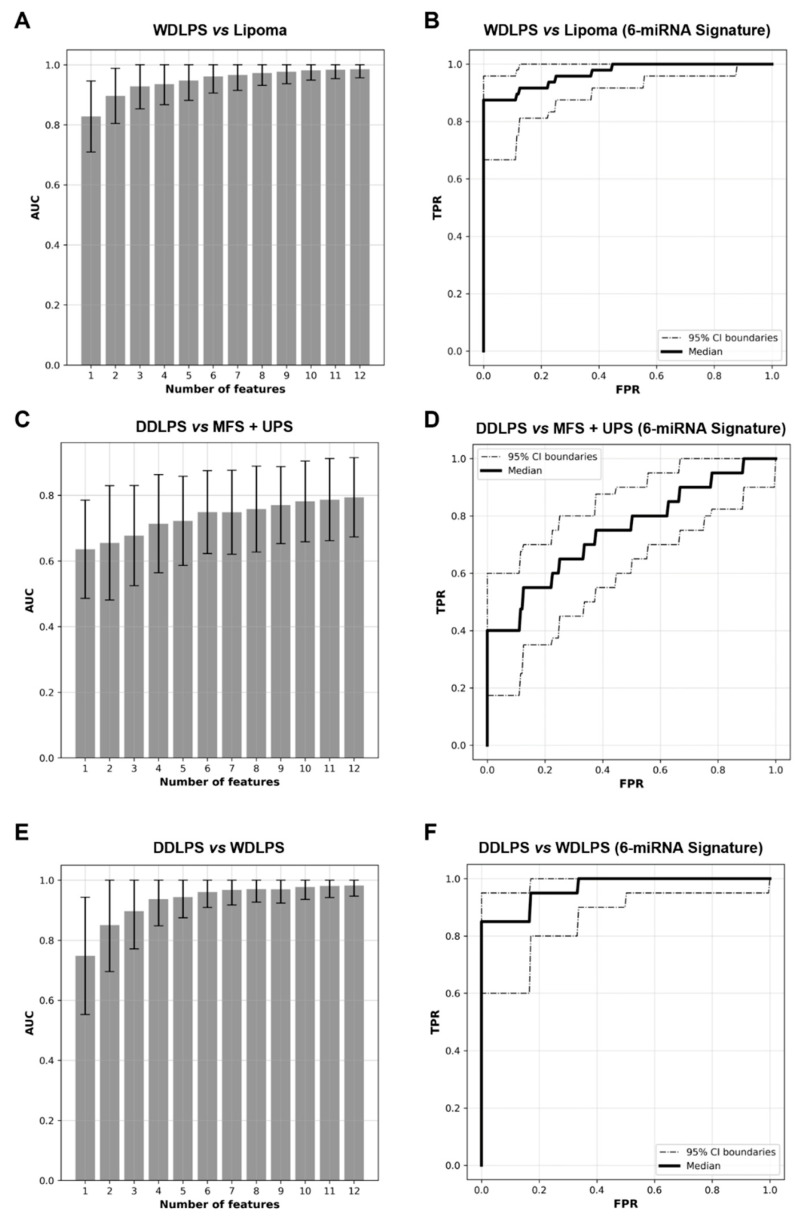
Accurate classification of soft tissue tumors using miRNA signatures. (**A**,**C**,**E**) Mean AUC (Area under the ROC curve) of miRNA signatures, with panels of 1 to 12 miRNAs, evaluated for accuracy in differentiating (**A**) WDLPS from lipoma, (**C**) DDLPS from MFS and UPS, (**E**) DDLPS from WDLPS. (**B**,**D**,**F**) ROC curves of the optimal miRNA panels evaluated for classifying (**B**) WDLPS from lipoma, (**D**) DDLPS from MFS and UPS, and (**E**) DDLPS from WDLPS.

To discriminate benign lipoma from morphologically similar malignant lipomatous tumors, miRNA signatures incorporating up to 12 features (differentially expressed miRNAs) were built to differentiate between lipoma (*n* = 34) and WDLPS samples (*n* = 24) (Figure 4A). A 6-miRNA panel would be optimal since AUC did not increase by more than 0.1 with the addition of more miRNAs to the panel. The optimal 6-miRNA classifier panel classified lipoma from WDLPS with an AUC of 0.963 (95% CI: 0.916 to 0.991) (Figure 4B). Among these six miRNA biomarkers, two were downregulated (miR-18a-5p and miR-769-5p) and four were upregulated (miR-196a-5p, miR-501-5p, miR-500a-5p, and miR-362-5p) in WDLPS samples compared to lipoma samples, respectively (Figure 4B).

### 2.4. miRNA Signature Can Distinguish DDLPS from Its Histologic Mimics

To investigate if miRNA biomarker panels can further separate dedifferentiated lipomatous tumors (i.e., DDLPS, which have a lower metastatic rate) from other more aggressive sarcoma mimics with spindle cell and/or pleomorphic features, miRNA expression profiles of DDLPS FFPE tissue samples (*n* = 20) were compared to that of MFS (*n* = 16) and UPS (*n* = 18). Of note, none of our MFS and UPS samples that had available FISH data showed evidence of *MDM2* gene amplification (Table 1). miRNA biomarker-classifier panels incorporating up to 12 features were then built and evaluated (Figure 4C). Our analysis showed that by using a signature of six miRNA features, a classification model can be established to separate DDLPS from MFS and UPS with AUC of 0.74 (95% CI: 0.64 to 0.82) (Figure 4D). Among the six features identified, miR-204-5p, let-7d-3p and miR-151a-3p were over-expressed while miR-516a-5p, miR-195-5p and miR-497-5p were under-expressed in DDLPS samples compared to that in MFS/UPS (Figure 4D).

### 2.5. WDLPS and DDLPS Are Associated with Distinct miRNA Expression Profiles

Beyond diagnostic utility, a comparison between the differentially expressed miRNA profiles between WDLPS and DDLPS may provide further insight into the molecular mechanisms involved in tumor progression and dedifferentiation in these tumors. Our analyses showed that miRNAs are significantly differentially expressed in WDLPS and DDLPS and that the two types of liposarcomas can be discriminated against using miRNA panels (Figure 4E). An optimal classifier built with six miRNAs distinguished WDLPS from DDLPS with an AUC of 0.96, (95% CI 0.89 to 0.99) (Figure 4F). Among these miRNAs, miR-21-5p, miR-15b-5p and miR-135b-5p were overexpressed, while miR-193a-5p, miR-423-5p and miR-191-5p were under-expressed in DDLPS compared to WDLPS. These miRNAs that are distinctly expressed in these tumor types may play an important role in the disease progression from WDLPS to DDLPS.

### 2.6. miRNAs Differentially Expressed between Lipoma and Liposarcoma, and between WDLPS and DDLPS

By analyzing the miRNA expression profiles of our various lipomatous tumors and their mimics, we also found that certain miRNAs are differentially expressed in the various entities. The most differentially expressed miRNAs are listed in Table 2 below.

### 2.7. Dysregulated Pathways in Liposarcomagenesis—Involvement of PI3K/AKT and MAPK Pathways

In an attempt to elucidate the molecular pathways that contribute to liposarcomagenesis and to gain some insight into the network that might be regulated by miRNAs, pathway analysis via gene set enrichment analysis (GSEA) was performed to identify differentially regulated pathways between different benign and malignant lipomatous subtypes. Using a *q*-value of 0.025, 18 pathways were found to be up-regulated in WDLPS compared to lipoma. The key components regulating many of these pathways include phosphoinositide 3-kinase (PI3K), protein kinase B (also known as Akt) and/or mitogen-activated protein kinase (MAPK) (Table 3). No differentially regulated pathways were found between WDLPS vs. DDLPS and DDLPS vs. MFS/UPS samples, indicating that the predominant pathways activated in these tumors are likely to be similar to each other.

## 3. Discussion

### 3.1. Ancillary Tests Currently Used in the Diagnosis of Adipocytic Tumors

Despite progress in the understanding of sarcoma pathobiology with the introduction of high-throughput genomics, novel diagnostic biomarkers based on gene expression profiling have yet to make a significant impact in the routine practice of most clinical centers. Although techniques such as FISH have been successfully incorporated into the diagnostic arsenal in many academic centers, they may still be beyond the resource constraints of smaller laboratories. Hence, it would be desirable to find alternative adjunctive ancillary tools that can aid in the diagnostic process.

Routine lineage-specific immunohistochemistry (IHC) has a limited role in the differential diagnosis of lipomatous tumors. While lineage-specific markers are relatively robust for the diagnosis of other soft tissue sarcomas, such as vascular markers for angiosarcomas and smooth muscle markers for leiomyosarcomas, S100 immunostain is only positive in adipocytes and not in the dedifferentiated component of liposarcomas.

Amplification of the chromosome 12q13-15 region involving the *MDM2* gene occurs in >95% of WDLPS and DDLPS cases [10,11,12], and identification of this genomic alteration by FISH [13] is used in most advanced medical institutions as an adjunctive diagnostic test. However, *MDM2* amplification can also be seen in other sarcomas [14,15], and hence interpretation of the results has to be done in the appropriate clinical context, especially when dealing with small biopsy samples. While amplification of the *MDM2* (and often *CDK4*) gene also leads to overexpression of the protein that IHC can detect, the available antibodies are difficult to optimize and have variable sensitivities that range from 50% to 100%. In addition, there exists inter-observer variability with regard to the threshold for classifying a case as positive [16]. While several studies have also suggested the addition of p16 immunostains to the panel of MDM2 and CDK4 immunostains to increase overall sensitivity, doing so conversely decreases the diagnostic specificity [16,17]. Hence, given that a significant proportion of IHC-negative cases still require FISH analysis, there is a limited diagnostic utility and economical benefit of using IHC alone.

### 3.2. miRNAs as Potential Diagnostic Biomarkers

In the recent decade, studies on the role of miRNAs have unveiled their promising potential as diagnostic biomarkers [9,18]. miRNAs play critical regulatory roles in many essential biological processes, and some have recognized the dysregulation of miRNAs as a fundamental hallmark of cancer [8,19]. Aberrant expression of miRNAs in tumors (so-called tumor miRNA signatures) makes them ideal candidates as tumor biomarkers [8,20,21]. Indeed, miRNAs have been shown to be helpful in tumor classification and subtyping in line with the cell of origin and differentiation state [22,23,24]. The ability of miRNA expression profiles in discriminating histologic subtypes of tumors has been successfully demonstrated for lung cancer [25,26,27], renal cell carcinoma [28,29,30], and mesothelioma [31]. Another attribute of miRNAs is their stability in biological specimens due to their small size and resistance to degradative processes. The high stability of miRNAs compared to other biomolecules, such as mRNA and DNA, enables their robust detection and quantification in meager amounts of materials (such as core needle biopsy or fine-needle aspiration samples) and in compromised clinical samples such as FFPE tissue samples [32,33], thus opening the window for them to serve as novel molecular diagnostic biomarkers for routine clinical practice.

While many studies have looked into the miRNA expression profiles of STT [34,35,36,37,38,39,40], few delved specifically into exploring the potential of miRNA as a tool to aid the morphologic diagnosis of STT. This study investigated the potential utility of miRNA expression signature as an adjunctive test to improve the differential diagnosis of well-differentiated and dedifferentiated lipomatous tumors. Specifically, we set out to identify differentially expressed miRNAs that could be used in the accurate classification between lipoma and morphologically overlapping WDLPS, and miRNA classifiers that could separate DDLPS from other common high-grade sarcomas. To evaluate the potential of miRNA expression profiles as diagnostic biomarkers, we performed miRNA profiling of 350 well-annotated miRNA candidates chosen based on their abundant and stable expression in various tissues and serum (unpublished data from MiRXES). We conducted our miRNA analyses on FFPE tissue samples of a cohort of well-characterized cases based on the current diagnostic criteria with the intent of translating our findings to practical clinical use. These cases have also been verified by qualified histopathologists with expertise in soft tissue pathology. With 58 FFPE tissue samples of lipoma and WDLPS, we derived an optimal 6-miRNA classifier that enables their separation with high confidence (AUC = 0.963). The unique miRNA expression signatures corresponding to benign lipoma and malignant WDLPS provide a proof-of-concept that miRNA expression profiling can be a powerful tool to discriminate well-differentiated lipomatous tumors of benign nature from malignant ones in routine clinical settings to aid in objective diagnostic decision-making, especially in the setting of small biopsy samples which often poses greater diagnostic difficulty. In addition, with miRNA expression profiling, we also identified a miRNA panel that can discriminate DDLPS from the more aggressive sarcoma mimics MFS and UPS using a 6-miRNA signature, with an AUC of 0.740.

Both FISH and miRNA analyses utilize FFPE tissue which is one of the most readily available tissue sources in clinical practice. However, FFPE tissue samples have their limitations as well. While it is known that the fixation and preservation processes involve the usage of chemicals that can degrade or alter the quality of nucleic acids preserved within the tissue, studies have shown that miRNAs, being small, have the advantage of remaining relatively stable despite these deleterious processes, possibly related to their association with RNA-induced silencing complex (RISC) [41]. Another known limitation of FFPE tissue samples is the degradation of the quality of nucleic acid over time. Nonetheless, Siebolts et al. found that the quality of miRNA extracted from FFPE tissue samples was not significantly reduced within 5–7 years, and was only significantly reduced in samples that were 10–20 years old [42]. As the aim is for miRNA analysis to be a potential diagnostic adjunct, its main usage would hence be on current/relatively fresh samples, and this issue would be less of a concern.

### 3.3. Biological Relevance of Differentially Regulated miRNAs

#### 3.3.1. miRNAs Differentially Expressed between Lipoma and Liposarcoma

Beyond diagnostic utility, analyses of dysregulated miRNAs in tumor samples may also shed light on biological relevance and contribute to further understanding of the mechanisms of liposarcoma tumorigenesis and progression. The top miRNAs that were found to be differentially expressed between benign lipoma and malignant liposarcoma in our study include miR-144-3p, miR-144-5p, miR-451a and miR-196a-5p. Our results showed that miR-144 and miR-451a were downregulated in WDLPS/DDLPS compared to lipoma. These findings were in concordance with several studies reporting the downregulation of miR-144/451a in multiple cancers [43,44,45,46,47,48], as well as in WDLPS and DDLPS [37,38,49]. Our findings are in keeping with a tumor suppressor-like function of miR-144 and miR-451a in adipocytic tissue along with these published findings. Furthermore, in line with our observation, miR-196a-5p was reportedly upregulated in cancers and shown to act as an oncogene by exerting multiple functions in tumorigenesis and cancer progression [50,51,52]. In osteosarcoma, elevated expression of miR-196a predicts poor prognosis [53] and has been shown to promote cell migration, invasion and the epithelial–mesenchymal transition by targeting HOXA5 [54]. To the best of our knowledge, no association of miR-196a-5p with the pathogenesis of liposarcoma has been reported so far. Our finding reveals a potential pathogenic role of miR-196a-5p in the malignant transformation of adipocytic tissue. Its inclusion in our optimal 6-miRNA signature for the classification of lipoma from WDLPS further shows its potential as a diagnostic biomarker. Future work on molecular targets regulated by miR-196a-5p may shed light on its involvement in other oncogenic mechanisms underlying liposarcomagenesis. The dysregulation of miRNAs in liposarcoma has also been investigated in a limited fashion by others. Other dysregulated miRNAs that have been reported include upregulation of miR-214, miR-199a, miR-155, miR-21, miR-26a and downregulation of miR-10b, miR-126, miR-143, miR-145, miR-1257, miR-193b and the let-7 family in WDLPS/DDLPS compared to benign fatty lesions [37,38,55,56,57,58,59]. In keeping with the results of these previously published studies, our data confirm the upregulation of miR-214-3p, miR-199a, miR-21-3p, miR-21-5p and downregulation of miR-10b, miR-126-3p, miR-126-5p, miR-143-3p, miR-143-5p, miR-145-5p, 193b-3p in WDLPS/DDLPS compared to benign lipoma, further supporting the biological significance of these miRNAs as validated biomarkers for WDLPS/DDLPS.

#### 3.3.2. miRNAs Differentially Expressed between WDLPS and DDLPS

To better understand the molecular changes undergone by WDLPS during progression, i.e., dedifferentiation to DDLPS, we also compared the miRNA expression profiles of these two biologically related but distinct tumor types. We found that miR-193a-5p and miR-423-5p were significantly downregulated while miR-21-5p, miR-454-3p, miR-374a-5p and miR-15b-5p were upregulated in DDLPS compared to WDLPS. Of these, the important role of miR-193a-5p as a tumor suppressor has previously been studied in hepatocellular carcinoma [60], breast cancer [61], non-small-cell lung cancer [62] and osteosarcoma [63]. Similarly, the tumor-suppressive function of miR-423-5p has also been demonstrated in ovarian cancer [64], colon cancer [65], nasopharyngeal carcinoma [66] as well as osteosarcoma [67]. miR-21 is an extensively studied miRNA that is widely overexpressed in multiple cancers, including sarcomas [68,69,70,71,72,73,74,75], while the upregulation of miR-374a-5p has been found in several cancers, including gastric cancer [76], breast cancer [77] and osteosarcoma [78]. Therefore, it is not surprising that we also found overexpression of miR-21 and miR-374a-5p in WDLPS samples compared to lipoma. However, the functions of miRNA-454-3p and miR-15b-5p appear to be complex, with both tumor suppressive or oncogenic functions being ascribed to the two miRNAs in different cancers, purportedly via different pathways [79,80,81,82,83,84,85,86,87,88]. Future studies are needed to explore the potential functional roles that these miRNAs may have in the dedifferentiation of WDLPS.

### 3.4. Potential Pathways Involved in Liposarcomagenesis

Given the divergent or convergent regulatory role miRNAs can exert on signaling pathways, we additionally performed gene enrichment analysis by GSEA on the differentially expressed miRNA between benign and malignant lipomatous tumors to investigate the dysregulated pathways involved in the malignant adipocytic transformation. In line with published studies, our analysis revealed high activation of PI3K/AKT/mTOR and MAPK pathways in liposarcoma compared to benign adipose tissue. The PI3K/AKT/mTOR pathway is a well-known survival pathway that is dysregulated in many cancers [89,90]. The role of this pathway in liposarcoma has been a focus of some previous studies. Using clinical samples and a zebrafish model, Gutierrez et al. [91] reported a central role for oncogenic AKT signaling in adipocyte transformation and implicating AKT as a potential therapeutic target in unresectable WDLPS/DDLPS. Another study reported activating PIK3CA mutations in about 14% of liposarcoma samples [92]. Furthermore, inhibition of the pathway and its downstream effector, mTOR, has also been found to induce apoptosis in liposarcoma cell lines [93]. DDLPS frequently bears additional genetic changes/recurrent mutations as compared to WDLPS, including 1p32 and 6p23 amplifications causing oncogenic overexpression of ASK1/MAP3K5, JUN, and activation of the MAPK pathway [10,57,94]. ERK, JNK, and p38 MAP kinase pathway activation have also been observed in transgenic mice with WDLPS development as a downstream response to increased interleukin-22 levels [95].

## 4. Materials and Methods

### 4.1. Sample Selection

Formalin-fixed paraffin-embedded (FFPE) tissue samples were obtained from the Department of Pathology, National University Hospital (NUH), Singapore. Prior to the study, Institutional Review Board approval (DSRB) was obtained for all samples in accordance with the NUH’s Institutional Review Board (IRB) Guidelines. Total FFPE tissue samples included: lipoma (*n* = 34), WDLPS (*n* = 24), DDLPS (*n* = 20), MFS (*n* = 16) and UPS (*n* = 18) (Table 4). All cases were reviewed by expert soft tissue pathologists to verify the diagnosis (based on histomorphologic diagnostic criteria delineated in the World Health Organization (WHO) Classification of Soft Tissue Tumors [2]) and to select the representative tissue areas for subsequent miRNA expression profiling in this study. The percentage of tumor cells was estimated to be at least 80–90% for each sample. 

### 4.2. Fluorescence In Situ Hybridization (FISH)

The majority of the WDLPS and DDLPS cases were diagnosed based on classical histomorphologic features or had prior *MDM2* fluorescence in situ hybridization (FISH) performed clinically for the diagnosis. For the MFS and UPS cases, tissue microarrays (TMAs) were constructed from the available tissue samples and the sections were subjected to *MDM2* FISH analysis where possible. TMA sections of 4 µm containing FFPE tissue cores were placed on electrostatically charged slides (Platinum Pro, Matsunami Glass Ind. Ltd., Kishiwada, Japan). FISH processing was done using the IntelliFISH Universal FFPE Tissue Pretreatment Kit (Vysis, Downer’s Drove, IL, USA) according to the manufacturer’s instructions and established laboratory protocol. The sections were then subjected to direct FISH using the Vysis MDM2/CEP 12 FISH Probe Kit (Vysis, Downer’s Drove, IL, USA). A total of 30 non-overlapping, intact interphase nuclei were scored per case using fluorescence microscopy. Only nuclei containing both red and green signals were enumerated. The ratio of *MDM2* signals to the Centromeric 12 probe signals was calculated. A ratio greater than or equal to 2.0 is interpreted as amplified and a ratio of less than 2.0 is non-amplified. The FISH images were obtained using an Olympus BX61 microscope and captured on the Applied Image Analysis System v.3.93 (Applied Imaging, Pittsburgh, PA, USA).

### 4.3. RNA Extraction and miRNA Profiling

miRNA profiling was carried out with a patented modified stem-loop mediated reverse transcription-quantitative polymerase chain reaction (mSMRT-qPCR) miRNA assay in a highly controlled workflow. Focusing on miRNAs strongly associated with various cancer types, 350 miRNAs were profiled and the process was carried out as follows. Total RNA was extracted from the FFPE tissues using the Qiagen miRNeasy FFPE kit (Hilden, Germany) on a semi-automated QiaCube system. A set of three spike-in control RNAs, all with sequences different from annotated mature human miRNAs (miRbase version21), was added to the sample lysis buffer prior to RNA isolation. The quantified levels of the spike-in control RNAs were used to normalize RNA isolation efficiency. Following isolation, the isolated miRNAs were reverse transcribed using miRNA-specific reverse transcription (RT) primers as per the manufacturer’s instruction (MiRXES). Concurrently, a 6-log serial dilution of synthetic templates for each miRNA and a non-template control (NTC) were reverse transcribed. All the sample and template cDNAs were then pre-amplified through a 14-cycle PCR reaction using Augmentation Primer Pools (MiRXES). Following cDNA amplification, a total of 350 miRNAs were measured, with technical replicates, via qPCR using miRNA-specific qPCR assays (MiRXES) on QS5 384-well qPCR system (Applied Biosystems).

### 4.4. Data Processing and Statistical Analysis

After the completion of the experiments, the raw threshold cycle (^) values were determined using the QS5 software with an automatic baseline setting and a threshold of 0.5. The absolute copy numbers of each miRNA were determined through interpolation of the C_t_ values to that of the synthetic miRNA standard curves and adjusted for RT-qPCR efficiency. Technical variations introduced during all experimental steps such as RNA isolation, reverse transcription, and qPCR were normalized using the spike-in control RNAs. Any miRNAs that were detected in less than 90% of all samples profiled were excluded from the subsequent analysis. All subsequent analyses were performed on normalized (via global mean normalization) and log2 transformed miRNA expression values.

### 4.5. Feature Selection for Tumor Classification

The following procedures were performed to identify miRNAs (features) that can best differentiate between (i) lipoma and WDLPS, (ii) WDLPS and DDLPS, and (iii) DDLPS and MFS/UPS.

From the full panel of 350 miRNAs, any miRNAs detected in less than 90% of all samples were removed, resulting in a final panel of 287 miRNAs. For these miRNAs, any non-detection or outliers were replaced by imputation. If the expression value of a miRNA in one sample is not detected, or the expression value is less than the mean by more than 3 standard deviations, it is replaced by the minimum value of that miRNA within 3 standard deviations of the mean. If the expression value is more than 3 standard deviations above the mean, it is replaced by the maximum value within 3 standard deviations of the mean.

A 4-fold cross-validation method, with 100 iterations, was used to select the features. The samples were split between the training and validation sets such that the ratio between the sample subtypes was similar. For each training set, the panel of 287 miRNAs was first transformed by standardization and was then fitted into a logistic regression model with an L2 penalty. miRNAs were sorted by their absolute coefficients and the top 1 to 12 miRNAs were chosen for model building. Support vector machines with a radial basis function kernel were used for classification and the prediction performance was tested on the validation set. The optimal number of markers was decided when the incremental of cross-validated AUC is smaller than 0.01 when additional markers were added. Using the results of all 100 iterations, the results were evaluated with receiver operating curves. Top markers were chosen by combining all data together and repeating the feature selection process with the optimal number of markers. All analysis was performed with Scikit-learn 1.0 in Python 3.8.12 (program downloadable from https://www.anaconda.com/products/distribution, accessed on 16 June 2022).

### 4.6. Receiver Operating Characteristic (ROC) Curve

The area under the receiver operating characteristic (ROC) curve, or AUC, was used to evaluate the diagnostic performance of the panel of selected features. For each 4-fold cross-validation, cross-validated ROC was plotted by averaging ROCs in each test set. One hundred repeats of such cross-validated ROCs were used to calculate 95% confidence intervals of the cross-validated ROCs. All analysis was performed with Scikit-learn 1.0 in Python 3.8.12.

### 4.7. Silico Prediction of the miRNA Regulatory Network

Pathway analysis was performed with all samples pooled together through the miRSEA method (MiRSEA: Discovering the pathways regulated by dysfunctional MicroRNAs). miRNA and mRNA linkages were curated with the miRTarBase Release 7.0 based solely on strong experimental evidence. The Molecular Signatures Database (MSigDB), Collection C2 (including Kegg, Reactome, Pathway Interaction Database and Biocarta), from the Broad Institute was then used to determine the involvement of different genes in each pathway in the database.

Briefly, the miRNA and the pathway were correlated together by identifying the specific strength of the miRNA targeting the pathway. A *p*-value for the miRNA-pathway association was calculated based on the hypergeometric distribution. The miRNA fold change together with the *p*-value for pathway targeting was used to calculate the regulation of the pathway with a weighted Kolmogorov–Smirnov-like statistic. Pathways targeted by less than 10 miRNAs or more than 200 miRNAs were ignored. In addition, if less than 50% of miRNA targeting a specific pathway was measured in the experiment, the pathway was also ignored. The *p*-value of any pathway regulation was calculated by randomly permutating samples 5000 times. FDR correction was carried out by using the null distribution of all pathways and the enrichment of the un-permutated data set. miRNAs in the leading edge were defined by selecting miRNA before the maximum F-score and targets corresponding to the leading edge miRNA were investigated. Pathways with *p* < 0.01 and false discovery rate q < 0.05 were considered significant. The analysis was performed with MatLab 2020.

## 5. Conclusions

Our results suggest that molecular analysis of miRNA expression profiles can provide additional information that complements conventional diagnostic methods. Specifically, we have identified a 6-miRNA panel that can accurately differentiate between benign lipoma and WDLPS, and a separate 6-miRNA panel that helps separate DDLPS from other common sarcoma subtypes. We believe that miRNA-based assays have a potential as complementary ancillary tools to aid pathologists in diagnosing these morphologically similar but biologically disparate lipomatous tumors. Further large batch study remains a necessary work to validate the promising results and optimize the biomarker panels.

## Figures and Tables

**Table 1:** Results of *MDM2* FISH analysis performed on MFS and UPS cases.

*MDM2* FISH Results	MFS (*n* = 16)	UPS (*n* = 18)
Amplified (positive)	0	0
Non-amplified (negative)	13	11
Data not available	3	7

**Table 2:** List of most differentially expressed miRNAs in our clinical samples.

	Upregulated	Downregulated
In liposarcoma (including WLDPS and DDLPS) compared to lipoma	miR-196a-5p	miR-144-3p miR-144-5p miR-451a
In DDLPS compared to WDLPS	miR-21-5p miR-454-3p miR-374a-5p miR-15b-5p	miR-193a-5p miR-423-5p

**Table 3:** Differentially regulated pathways between lipoma and WDLPS.

Pathway	Key Components	*p*-Value	*q*-Value
Cardiac EGF pathway	GPCR, Phospholipase C, PKC Delta, MAPK	1.00 × 10^−4^	1.49 × 10^−2^
Caspase pathway	Caspase cascade	1.00 × 10^−4^	1.49 × 10^−2^
AMB2 neutrophils pathway	PI3K, Akt, MAPK	1.00 × 10^−4^	1.49 × 10^−2^
RAP1 signaling	PI3K, Akt, MAPK	1.00 × 10^−4^	1.49 × 10^−2^
Insulin glucose pathway	PI3K, Akt	1.00 × 10^−4^	1.49 × 10^−2^
PI3KCI AKT pathway	PI3K, Akt	2.00 × 10^−4^	2.13 × 10^−2^
PKB mediated events	Akt	2.00 × 10^−4^	2.13 × 10^−2^
Integrin alphaIIB beta3 signaling	thrombin, ADP, collagen, fibrinogen and thrombospondin, PTK2/FAK	3.00 × 10^−4^	2.49 × 10^−2^
Costimulation by the CD28 family	CD28, CTLA4, ICOS, PD1 and BTLA receptors, PI3K, Akt	4.00 × 10^−4^	2.49 × 10^−2^
CD40 pathway	JAK/STAT, NF-kappaB, MAPK, P38, JNK	4.00 × 10^−4^	2.49 × 10^−2^
Regulation of the actin cytoskeleton by Rho GTPases	Rho, ROCK	4.00 × 10^−4^	2.49 × 10^−2^
P38 MAPK pathway	MKK3, MKK6, P38	5.00 × 10^−4^	2.49 × 10^−2^
IL2 PI3K pathway	IL2, PI3K, Akt	5.00 × 10^−4^	2.49 × 10^−2^
Lysophospholipid pathway	Rho, PI3K, Akt, MAPK, PLC, cAMP	5.00 × 10^−4^	2.49 × 10^−2^
Telomerase pathway	ATM, ATR, TERT, Akt	5.00 × 10^−4^	2.49 × 10^−2^
Chemical pathway	cytochrome c, AIF, caspase cascade	6.00 × 10^−4^	2.49 × 10^−2^
ECM regulators	Components Regulating remodeling of the extra-cellular matrix	6.00 × 10^−4^	2.49 × 10^−2^
HNF3B pathway	Regulated by PI3K, MAPK. Regulates cancer	6.00 × 10^−4^	2.49 × 10^−2^

**Table 4:** Summary of demographics and characteristics of patient samples used.

	Lipoma (*n* = 34)	WDLPS ^1^ (*n* = 24)	DDLPS ^2^ (*n* = 20)	MFS ^3^ (*n* = 16)	UPS ^4^ (*n* = 18)
**Gender**					
Male	23	17	13	5	10
Female	11	7	7	11	8
**Age** (median, range)	51.5 (5–81)	59 (42–87)	64 (39–85)	67 (36–94)	60.5 (26–94)
**Race**					
Chinese	25	15	14	15	12
Malay	4	3	3	1	2
Indian	2	0	0	0	0
Others ^5^	3	6	3	0	4
**Anatomic site**					
Extremities ^6^	3	6	6	9	8
Central body sites ^7^	24	16	13	5	10
Others ^8^	7	2	1	2	0

^1^ WDLPS = well-differentiated liposarcoma; ^2^ DDLPS = dedifferentiated liposarcoma; ^3^ MFS = myxofibrosarcoma; ^4^ UPS = undifferentiated pleomorphic liposarcoma; ^5^ Others include Caucasian, other Asian ethnicities, and samples with race not specified; ^6^ Extremities include upper and lower limbs; ^7^ Central body sites include retroperitoneum, abdominal cavity, thoracic cavity, inguinal canal, abdominal wall and chest wall; ^8^ Others include head and neck sites and skin (site not otherwise specified).

## Data Availability

Publicly available datasets were used in this study. The datasets can be found here: https://www.mirbase.org/ (miRBase), accessed on 16 June 2022; https://mirtarbase.cuhk.edu.cn/~miRTarBase/miRTarBase_2022/php/index.php (MiRTarBase), accessed on 16 June 2022; https://www.gsea-msigdb.org/gsea/msigdb/ (MSigDB), accessed on 16 June 2022.
